# Ablation of premature ventricular contraction triggering ventricular fibrillation in a patient with early repolarization syndrome

**DOI:** 10.1111/anec.12792

**Published:** 2020-08-17

**Authors:** Liu Qifang, Huang Jing, Zhao Yidong, Yang Long

**Affiliations:** ^1^ Cardiology Guizhou Provincial People's Hospital Guiyang China

## Abstract

Early repolarization syndrome is associated with an increased risk of arrhythmic death caused by ventricular fibrillation (VF). VF is usually initiated by premature ventricular contractions (PVCs), and PVCs commonly arise from Purkinje system, the ventricular outflow tract, and papillary muscles. We report the case of a patient with J wave syndromes and recurrent VF, triggered by PVCs originating from the tricuspid annular region. VF was successfully suppressed by catheter ablation of the triggering PVCs, and there has been no recurrence of VF during a follow‐up period of 6 months.

## 1 INTRODUCTION

Ventricular fibrillation (VF) is the most serious arrhythmia associated with sudden cardiac death. In some patients with no apparent structural heart disease, the underlying mechanism and etiology for the arrhythmia cannot be identified despite an extensive investigation. Reports suggest there is a clinical relationship between early repolarization (ER) and VF in patients without structural heart disease (Haissaguerre, Nademanee, Hocini, Cheniti, et al., 2019). Prior works confirmed VF can be suppressed by catheter ablation of the triggering premature ventricular contractions (PVCs) originating from Purkinje system, the right ventricular outflow tract, and papillary muscles (Anderson et al., 2019; Nakamura et al., 2019). We reported a case of idiopathic VF with PVCs rarely originating in the tricuspid annular region and ER in the inferolateral leads.

## 2 CASE PRESENTATION

**Figure 1:**
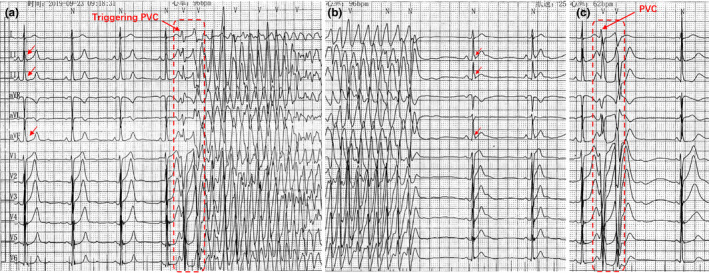
Electrocardiograms showing early repolarization and ventricular fibrillation

A 28‐year‐old man was referred to our hospital for palpitation and a history of syncope. No cardiac risk, drug abuse, or family history of sudden cardiac death was found. The patient's cardiac evaluation during admission included echocardiography, delayed gadolinium‐enhanced MRI, and coronary angiography, which demonstrated no original structural heart disease. Routine 12‐lead electrocardiogram (ECG) presents significant J‐ST elevation in the inferior‐lateral leads and PVCs with left bundle branch block. A Holter ECG documented 17,854 PVCs and non‐sustained VF. Holter ECG monitoring revealed that the QRS morphology of the first beat in VF episode was identical to that of recorded PVCs. Interestingly, after an episode of VF, the QRS complexes showed clear accentuation of ER, as compared with baseline (Figure 1).

**Figure 2:**
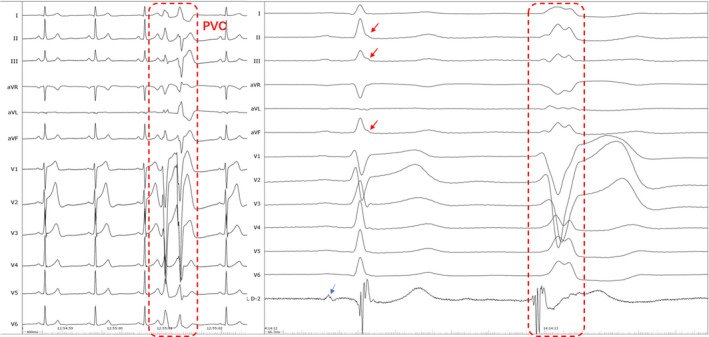
Intracardiac electrograms exhibiting the premature ventricular contrations and the successful ablation site

**Figure 3:**
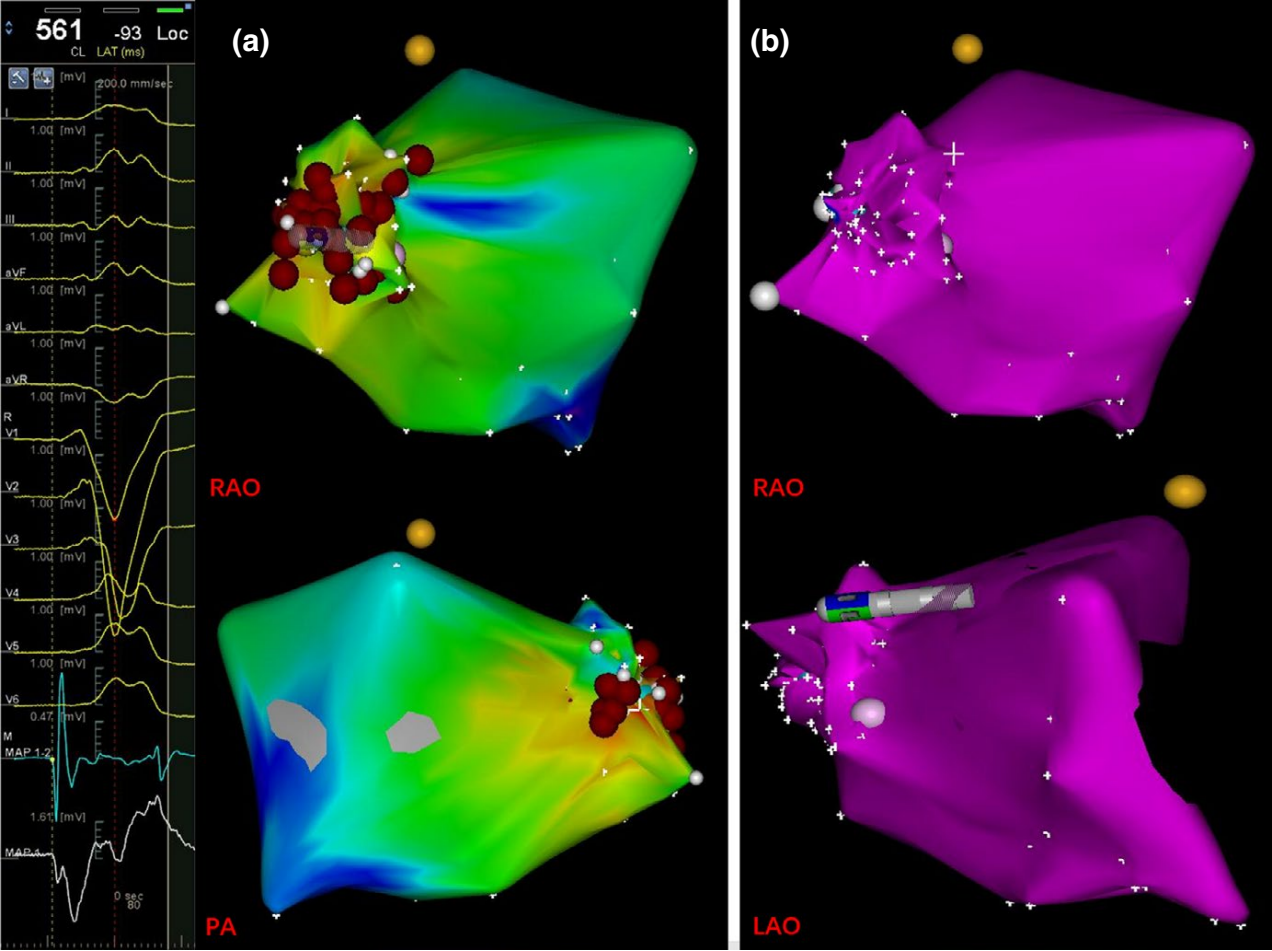
CARTO electroanatomical mapping

The episodes of VF and PVCs could not be suppressed by different drugs such as amiodarone, metoprolol at another hospital. Electroanatomical mapping and ablation were performed using a 3.5‐mm‐tip open irrigated catheter (Thermocool, Biosense Webster, Diamond Bar, CA, USA) with the CARTO mapping system (CARTO3, Biosense Webster). During electrophysiological study, no ventricular tachycardia and VF were induced, but frequent PVCs were present and allowed us to perform activation sequence mapping. At the site of tricuspid annulus, the local activation time was recorded for 36 msec prior to the earliest start of the QRS complexes (Figure 2). No Purkinje‐like and discrete potential preceding the QRS and fractionated electrograms were recorded at or around the earliest activation site of the tricuspid annulus. The normal local voltage was observed in the successful ablation regions compared with the other part of the ventricular by voltage maps in sinus rhythm (Figure 3). Radiofrequency ablations (power of 30 W, temperature cutoff of 43°C) were delivered at the site with the earliest activation and led to complete elimination of PVCs. Additional RF lesions were applied surround the successful ablation site. PVCs and VF were not inducible by isoproterenol or programmed electrical stimulation. During a follow‐up period of 6 months, no arrhythmic recurrences were recorded on 24‐hour Holter ECG monitoring, and the patient remained free of syncope and palpitation without medications.

## 3 DISCUSSION

To the best of our knowledge, we firstly report a case that radiofrequency ablation for VF‐triggering PVCs originating from the tricuspid annular area successfully suppressed the recurrence of VF in a patient with ER syndrome. Recent studies demonstrated that J wave is related to ER abnormalities or delayed depolarization owing to microstructural alterations (Haissaguerre, Nademanee, Hocini, Duchateau, et al., 2019). In this case, the abnormal electrograms related to microstructural alterations were not recorded in the endocardium of the right ventricle. Electrophysiologic mapping in the epicardium was not performed. However, it was possible that PVCs were a bystander located in the tricuspid annulus region as the trigger of the VF and depolarization or repolarization abnormality underlying inferolateral J wave syndrome was responsible for maintenance of VF. Accordingly, the suppression of the VF was achieved with catheter ablation of the triggering PVCs. However, the underlying mechanisms that initiate and sustain VF are still poorly understood, and further studies are needed to evaluate the accuracy of such a mechanism.

## 4 CONCLUSION

Ventricular fibrillation is a lethal arrhythmia that may present in patients without structural heart disease. Catheter ablation of triggering PVCs is recognized as a reasonable therapy when the clinical‐triggering PVCs are high burden. Although suppression of VF can be achieved by the elimination of triggering PVCs, long‐term follow‐up is needed. Furthermore, ablation of VF substrates is effective in treating symptomatic ER syndrome patients (Nademanee et al., 2019). Radiofrequency ablation may be considered for treatment of VF‐triggering PVCs to minimize the risk of recurrent of VF in patients with J wave syndrome.

## CONFLICT OF INTEREST

The authors declare that they have no conflict of interest.

## AUTHOR CONTRIBUTIONS

Dr Liu undertook the electrophysiology procedure, obtained the Figures and drafted the manuscript. Dr Huang and Dr Zhao wrote portions of the text. Dr Yang undertook the electrophysiology study and revised the manuscript.

## ETHICS

The reported study has received written consent as required by the appropriate ethics committee.
